# Comparison of the Effects of Kinesio Taping and Foam Roller Exercises in Individuals with Non-Specific Chronic Low Back Pain

**DOI:** 10.3390/healthcare14172853

**Published:** 2026-09-04

**Authors:** Mustafa Emre Cicekler, Hazal Genc, Gamze Demircioglu, Kevser Burma

**Affiliations:** 1 Department of Physiotherapy and Rehabilitation, Institute of Health Sciences, Bahçeşehir University, Istanbul 34353, Türkiye; ciceklermustafaemre@gmail.com; 2 Physiotherapy Program, Department of Therapy and Rehabilitation, Arnavutköy Darülaceze Vocational School of Health Services, University of Health Sciences, Istanbul 34277, Türkiye; hazaloksuz@gmail.com; 3 Department of Physiotherapy and Rehabilitation, Faculty of Health Sciences, Bahçeşehir University, Istanbul 34353, Türkiye; 4 Department of Occupational Therapy, Faculty of Health Sciences, Istanbul Atlas University, Istanbul 34408, Türkiye; gamze.demircioglu@atlas.edu.tr; 5 Department of Physiotherapy and Rehabilitation, Faculty of Health Sciences, Istanbul Atlas University, Istanbul 34408, Türkiye

**Keywords:** pain measurement, rehabilitation, foam roller, Kinesio taping, functional disability, low back pain

## Abstract

**Background/Objectives:** Non-specific chronic low back pain is a prevalent musculoskeletal disorder that impairs flexibility, sleep quality, functional capacity, and quality of life. Conservative treatments such as Kinesio taping and foam roller exercises are widely used, yet comparative evidence remains limited. This study aimed to compare their effects on pain, disability, flexibility, sleep quality, and kinesiophobia in individuals with non-specific chronic low back pain. **Methods:** Sixty patients were randomly assigned to Kinesio taping or foam roller groups. The Kinesio taping group received lumbar taping twice weekly, while the foam roller group performed foam roller exercises for 5 min per session over a 6-week intervention period. Outcomes included the Visual Analogue Scale, Oswestry Disability Index, Pressure Pain Threshold, Pittsburgh Sleep Quality Index, Schober Test, Tampa Scale for Kinesiophobia, and Fingertip-to-Floor Distance. **Results:** Both groups showed significant improvements in pain intensity, disability, pressure pain threshold, and flexibility (*p* < 0.001). Sleep quality (*p* = 0.012) and kinesiophobia (*p* = 0.003) improved significantly only in the Kinesio taping group. Between-group comparisons showed lower post-treatment pain intensity in the foam roller group (*p* = 0.015) and lower kinesiophobia in the Kinesio taping group (*p* = 0.009). The between-group mean difference in pain change was −0.86 cm (95% CI: −1.57 to −0.15). No significant between-group differences were observed for the other outcomes. **Conclusions:** Both interventions improved pain and physical outcomes, with different response patterns. Foam roller showed more favorable findings for pain intensity, whereas Kinesio taping showed more favorable findings for kinesiophobia, with no between-group difference in sleep quality.

## 1. Introduction

Non-specific low back pain is a common musculoskeletal condition in which pain cannot be attributed to a specific disease or structural cause and accounts for approximately 90% of low back pain cases [1]. Low back pain is the leading cause of disability worldwide, affecting approximately 619 million people globally in 2020 [1]. When non-specific low back pain becomes chronic, typically persisting or recurring for more than 12 weeks [2], it may be associated with persistent pain, impaired physical function, psychological distress, and reduced quality of life. In addition to its impact on individuals, chronic non-specific low back pain contributes to a substantial societal and economic burden, including productivity loss [3].

In recent years, significant advancements have been made in managing low back pain through a range of conservative treatment approaches. Among these, myofascial release techniques such as foam rolling and kinesio taping have gained attention as non-invasive modalities with potential benefits for individuals with non-specific low back pain. Foam rolling facilitates the relaxation of myofascial tissues, enhances circulation, and supports recovery, with studies reporting improvements in muscle stiffness, flexibility, and functional capacity [4,5]. Kinesio taping, in turn, supports muscular activation, alleviates pain, and promotes lymphatic flow by stimulating subcutaneous tissues, thereby enhancing proprioception and neuromuscular coordination [6]. Both techniques are thought to influence not only local tissue responses but also engage peripheral and central mechanisms of pain modulation, including cutaneous mechanoreceptor activation and descending inhibitory pathways [7,8,9]. In addition, emerging evidence suggests that these interventions may have a regulatory effect on systemic symptoms commonly reported in individuals with chronic non-specific low back pain, such as fatigue, sleep disturbances, and stress-related dysregulation [10,11,12].

Despite these promising findings, evidence regarding the clinical effects of foam rolling and Kinesio taping in individuals with chronic non-specific low back pain remains limited. Previous studies have primarily investigated these interventions separately, including roller-based myofascial release and Kinesio taping in individuals with non-specific low back pain [13]. However, direct comparative evidence between these two approaches in this clinical population remains limited. Consequently, it remains unclear whether one approach provides greater benefit for clinically relevant outcomes such as pain, disability, mobility, and fear of movement. Furthermore, sleep disturbance and psychological factors are recognized as relevant components of the broader burden of chronic low back pain [14,15,16,17], supporting the assessment of outcomes beyond pain and physical function alone.

Therefore, the present study aimed to directly compare the effects of foam rolling and Kinesio taping, when combined with the same home exercise program, on pain intensity, functional disability, flexibility, sleep quality, and fear of movement in individuals with chronic non-specific low back pain. By evaluating both physical and patient-reported outcomes, this study aimed to provide direct comparative evidence regarding these two conservative interventions.

## 2. Materials and Methods

### 2.1. Participants

The International Statistical Classification of Diseases and Related Health Problems, 10th Revision, defines low back pain under the code M54.5 within the dorsopathy category. This condition is characterized by pain localized in the lumbar region, typically without an identifiable structural cause, and is commonly referred to as non-specific low back pain. Participants were recruited from the Bahçeşehir Healthy Life Center in Istanbul, Türkiye, following a clinical presentation of persistent low back pain. All had received a diagnosis of chronic non-specific low back pain from specialists in orthopedics or physical medicine and rehabilitation, based on symptom duration exceeding 12 weeks, clinical examination, and the exclusion of specific spinal pathologies and neurological deficits.

Inclusion criteria comprised: age between 20 and 60 years; persistent lumbar pain for at least 12 weeks; a pain intensity score greater than three on the Visual Analog Scale, representing at least moderate pain severity [17]; a sufficient cognitive and communicative ability to follow instructions; no contraindications to physical activity; no prior spinal surgery; and voluntary participation with signed informed consent.

Exclusion criteria were: presence of neurological or sensory impairments; history of spinal tumors, infections, fractures, or dislocations; previous cervical or lumbar operations, including disc-related procedures; recent radiofrequency ablation or corticosteroid injections within the past three months; allergy to kinesiology tape; diagnoses of severe osteoporosis, malignancy, systemic or inflammatory diseases; pregnancy; illiteracy; missing three consecutive treatment sessions; diagnosis of fibromyalgia or cervical disc herniation; development of any complications during or after the intervention; failure to meet the diagnostic criteria for chronic pain; or adherence to less than 75% of the prescribed home exercise program during the intervention period.

### 2.2. Ethical Committee Statement

This study was designed as a single-masked, randomized controlled trial with a two-group structure over a six-week intervention period. Following the ethics committee approval dated 19 April 2023 (decision number: 2023-08/06), the study was initiated and conducted between July and September 2024. The research was carried out in accordance with the principles of the Declaration of Helsinki and was registered on ClinicalTrials.gov with the registration number NCT06417255.

Before initiating the treatment program, all participants were thoroughly informed, both verbally and in writing, about the purpose of the study, the evaluation procedures, and the interventions to be administered. Written informed consent was obtained from all participants who voluntarily agreed to participate.

In this study design, outcome assessors were blinded to group allocation to reduce the risk of detection bias. Due to the nature of the intervention, neither the participants nor those administering the exercise programs could be blinded.

### 2.3. Randomization Process

Participants were randomly assigned to one of the two intervention groups using a computer-based random number generator (www.random.org). Randomization was performed after baseline assessments had been completed to ensure allocation concealment. Group assignments were recorded by a researcher not involved in the assessment process, and outcome assessors remained blinded to group allocation throughout the study to minimize detection bias.

**Figure 1:**
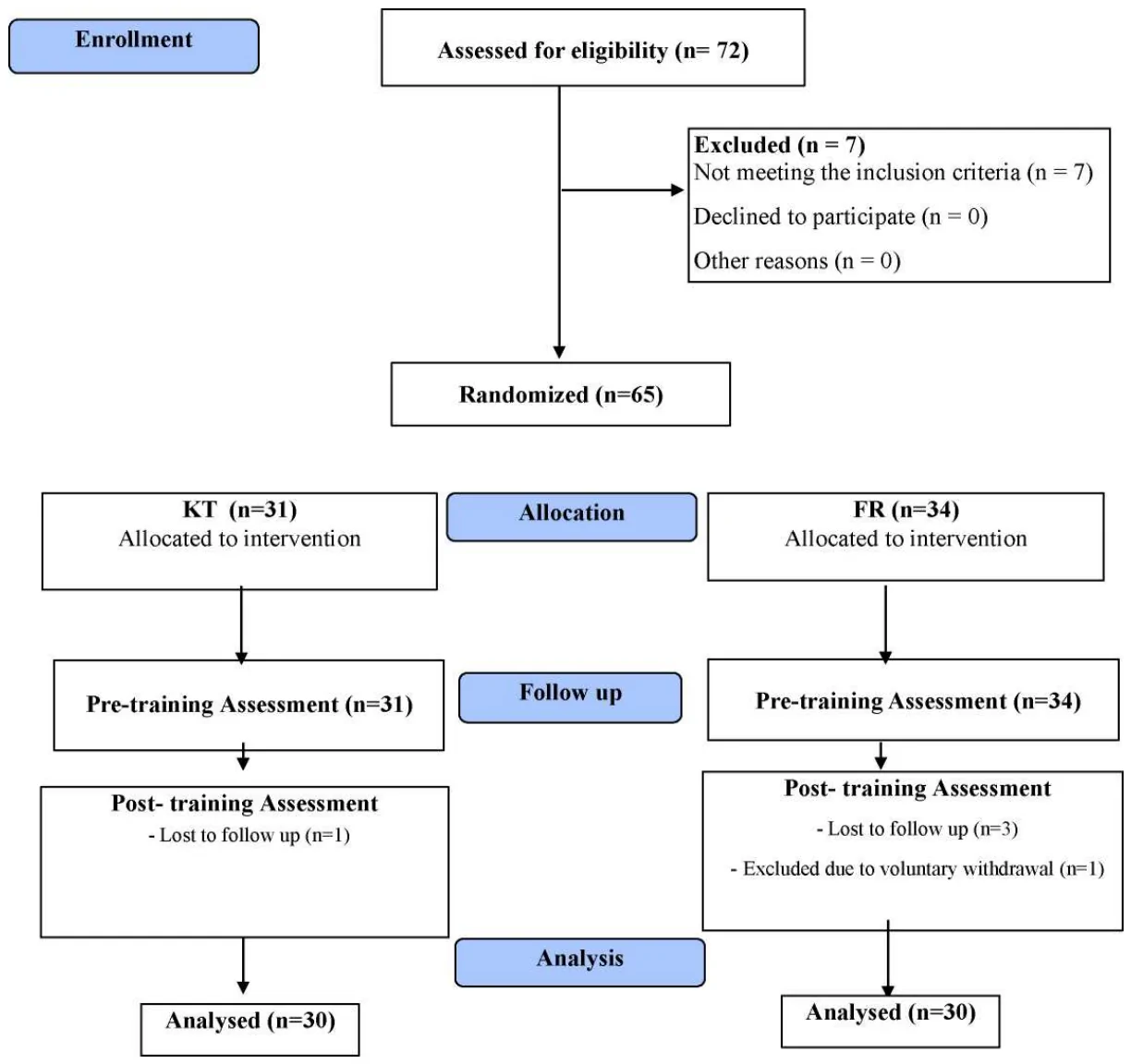
Consolidated standards of reporting trials (consort) flow chart for trial recruitment.

A total of 72 individuals were assessed for eligibility, of whom seven did not meet the inclusion criteria. The remaining 65 participants were randomly assigned to the Kinesio taping group (*n* = 31) or the foam roller group (*n* = 34). During the intervention period, one participant in the Kinesio taping group did not complete the follow-up. In the foam roller group, three participants did not complete the follow-up and one participant voluntarily withdrew from the study. As a result, 60 participants, with 30 in each group, completed the study and were included in the final analysis. Participant flow is presented in Figure 1 in accordance with the CONSORT 2025 reporting guideline, see Supplementary Materials [18].

### 2.4. Procedure

One group of the study participants received Kinesio tape while the other group was treated with foam rollers. Participants were randomly assigned to either the Kinesio taping group or the foam roller group using a computer-based random number generator (www.random.org). Both groups were given the same home exercise program without additional stimulation before, during, or after the exercises. Participants were instructed to perform the exercises thrice daily [19], completing ten repetitions each time. The home-based exercise program was progressively advanced over the 6-week intervention period while maintaining the same exercise frequency and number of repetitions. During weeks 1–2, the program consisted of basic isometric core stabilization exercises, stretching of the hamstrings, hip flexors, and lumbar extensors, lumbar mobilization exercises, and basic bridging. During weeks 3–4, exercise difficulty was increased by progressing the stabilization and bridging exercises and introducing bird-dog and partial curl-up exercises. During weeks 5–6, more advanced variations in the stabilization, bridging, and trunk-strengthening exercises were performed. Exercise progression was individualized according to each participant’s tolerance and ability to perform the exercises with appropriate technique. Isometric trunk exercises, such as abdominal bracing and plank variations, have significantly enhanced spinal stability and reduced pain in individuals with chronic non-specific low back pain [20]. Similarly, stretching and mobilization techniques have demonstrated efficacy in improving lumbar flexibility and mitigating muscular tightness, contributing to functional improvement and reduced disability [21]. Bridging and active trunk strengthening exercises, including bird-dog and partial curl-up movements, have been associated with enhanced neuromuscular control and endurance of core musculature, further supporting spinal health in chronic pain populations [22,23].

**Figure 2:**
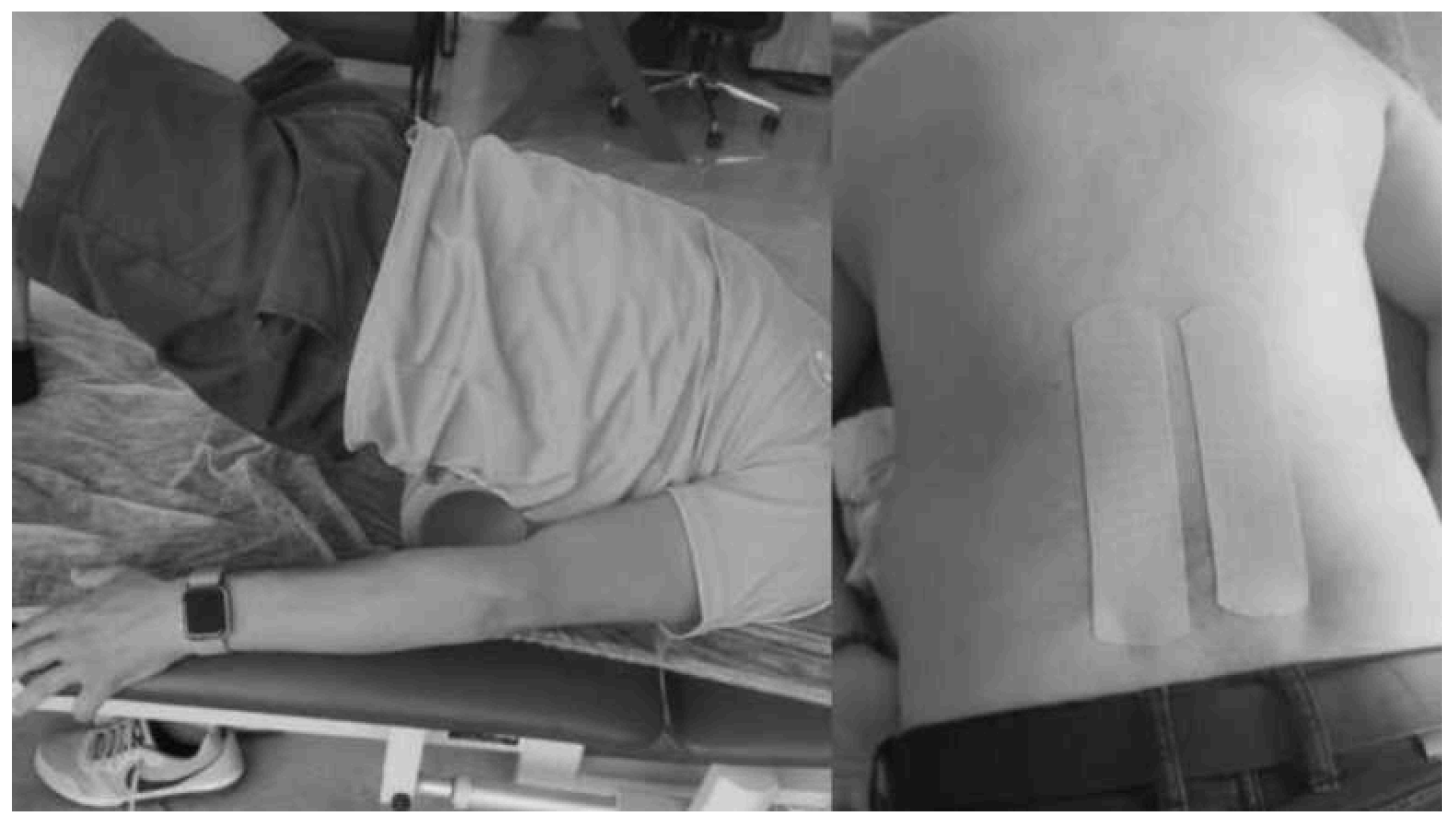
Foam Rolling and Kinesio Taping Applications.

According to the treatment protocols, participants in the kinesio taping group received applications of 5 cm wide standard KinesioTex Gold tape (kinesiotape-X050; Kinesio Tex, Tokyo, Japan) to the lumbar region twice a week. In the Kinesio taping inhibition technique applied to the lumbar region, two I-strips were placed horizontally at and above the level of the posterior superior iliac spine over the tense lumbar extensor muscles, with approximately 35–40% stretch [24]. In line with the original Kinesio Taping method described by Kase et al., each tape application remained on the skin for 3 to 5 days before being replaced. This regimen was maintained throughout the intervention to ensure continuous therapeutic stimulation [6]. A physiotherapist administered the taping with 7 years of clinical experience and international Kinesio taping certification. Outcome assessors remained blinded to group allocation throughout the study; however, participants and treating physiotherapists could not be blinded due to the nature of the interventions (Figure 2).

Participants in the foam roller group received applications with serrated Actifoam foam rollers (15 × 90 cm; Atermit, Gebze, Türkiye) for 5 min per session, twice weekly, over a 6-week intervention period. The session duration was selected within the range of short-duration, repeated foam rolling applications reported in the literature, where rolling is commonly performed in multiple sets of approximately 30–120 s [25]. A 6-week intervention period was also consistent with previous roller-based myofascial release research in individuals with non-specific low back pain [13]. Because Kinesio taping and foam rolling represent inherently different modes of application, intervention exposure was standardized according to the modality-specific protocol rather than matched by application duration. To identify the direction of restriction, a physiotherapist palpated the soft tissue and guided the participants accordingly. During each session, participants applied the foam rolling technique themselves under the supervision of a physiotherapist. Once the tissue barrier was detected, the fascial complex was targeted using controlled pressure without sliding over the skin or excessively compressing the tissue, within the standardized 5-min session (Figure 2).

Following the 6-week intervention period, all outcome measures were reassessed and recorded using the same procedures as in the pre-intervention assessment. Adherence to the home exercise program was monitored using daily exercise logs, which were reviewed weekly by the supervising physiotherapist. A minimum adherence rate of 75% was required for inclusion in the final analysis.

### 2.5. The Outcome

Our study randomly divided participants into two groups using random.org: the Kinesio taping and foam roller groups. Both groups were assessed before the treatment protocol and at the end of the six-week program to evaluate the intervention outcomes. The primary outcome measure was the Oswestry Disability Index, which assesses functional limitation in daily living activities [26]. Secondary outcome measures included the Pressure Pain Threshold, measured using a pressure algometer to assess soft tissue sensitivity [27], the Pittsburgh Sleep Quality Index to determine sleep quality over the past month [28], the Schober Test [29] to assess lumbar spine flexibility, the Tampa Kinesiophobia Scale [30] to measure fear of movement and re-injury, and the Fingertip-to- Floor Distance Test [31] to assess general body flexibility and specifically the mobility of the lumbar and hamstring muscles.

#### 2.5.1. Oswestry Disability Index

The Oswestry Disability Index assessed disability due to low back pain. It consists of 10 items addressing daily living activities, each scored from 0 to 5. Higher scores indicate greater disability. The Turkish version has been validated with excellent test–retest reliability (intraclass correlation coefficients = 0.91) [26].

#### 2.5.2. Visual Analog Scale

Pain intensity was measured using the Visual Analog Scale, a 10-cm horizontal line ranging from “no pain” to “worst imaginable pain.” Participants marked the point that best represented their current pain level. The Visual Analogue Scale is a reliable and straightforward instrument (intraclass correlation coefficients: 0.82) for assessing musculoskeletal pain intensity in both clinical settings and research. It is widely used and has demonstrated strong validity and reliability. The Turkish version has also been validated [32]. The minimum clinically significant difference for Visual Analogue Scale is 1.4 cm. The Visual Analogue Scale assessment, which takes approximately 1–2 min to administer, was utilized twice in the study at 6-week intervals [33].

#### 2.5.3. Pressure Pain Threshold

Pressure Pain Threshold was measured using a pressure algometer (Baseline® Dolorimeter, 12-1440; Fabrication Enterprises, Inc., White Plains, NY, USA) applied perpendicularly to pre-identified myofascial trigger points. Participants indicated when pressure turned into pain. This method is a valid and reliable way to assess mechanical pain sensitivity [33]. In this study, Pressure Pain Threshold measurements were performed bilaterally over the lumbar paraspinal muscles at the L3–L5 level, which are commonly evaluated in individuals with non-specific low back pain [34,35]. The intrarater reliability of pressure pain threshold measurements using an algometer is excellent, with intraclass correlation coefficients ranging from 0.91 to 0.97 [36].

#### 2.5.4. Schober Test

The Schober Flexibility Test was performed by measuring the distance between two marks on the lumbar spine before and during forward flexion, with a gain of less than 5 cm suggesting limited lumbar mobility; excellent intra- and inter-rater reliability has been demonstrated for this test (intraclass correlation coefficients = 0.95) [29,37].

#### 2.5.5. Fingertip-to-Floor Distance Test

This test evaluated flexibility, particularly of the hamstrings and lumbar spine, by recording the vertical distance between the fingertips and the floor when participants stood with knees extended and bent forward. It has shown excellent reliability (intraclass correlation coefficients ≈ 0.99) in clinical settings [38].

#### 2.5.6. Pittsburgh Sleep Quality Index

The Pittsburgh Sleep Quality Index was used to assess sleep quality over the past month across seven domains. A total score above 5 indicates poor sleep quality. The Turkish version has demonstrated good test–retest reliability with intraclass correlation coefficients values ranging from 0.82 to 0.87 [28].

#### 2.5.7. Tampa Scale of Kinesiophobia

Kinesiophobia was measured using the 17-item Tampa Scale of Kinesiophobia. Each item is scored on a 4-point Likert scale. Higher scores indicate a higher level of fear-avoidance. The Turkish version of the Tampa Scale for Kinesiophobia has shown excellent test–retest reliability with an intraclass correlation coefficient of 0.806 [30,39].

### 2.6. Statistical Analysis

Statistical analyses were performed using IBM SPSS Statistics Version 26 (IBM Corp., Armonk, NY, USA). Normality was assessed using the Kolmogorov–Smirnov test. Continuous variables were expressed as mean ± standard deviation (SD), and categorical variables as frequencies and percentages. Between-group comparisons of continuous variables were performed using independent-samples Student’s *t*-tests, while within-group pre- and post-treatment comparisons were performed using paired-samples Student’s *t*-tests. Categorical variables were compared using the chi-square test. Change scores (Δ) were calculated as post-treatment minus baseline values, and between-group differences in change scores were analyzed using independent-samples Student’s *t*-tests and reported with 95% confidence intervals (CIs). Statistical significance was set at *p* < 0.05. The final analyses included participants who completed the post-intervention assessment and met the predefined adherence criterion (≥75%). An intention-to-treat analysis was not performed.

The sample size was calculated using G*Power software (version 3.1.9.4; Heinrich Heine University Düsseldorf, Düsseldorf, Germany) based on an effect size (Cohen’s d = 0.70) derived from the Oswestry Disability Index results reported by Ozsoy et al. [13]. The required sample size was determined using a significance level of 0.05 and a statistical power of 0.95. To account for an expected 20% dropout rate, 72 participants were initially assessed for eligibility, and 60 participants completed the study and were included in the final analysis.

## 3. Results

The foam roller group and the Kinesio taping group each consisted of 30 participants. Mean adherence to the home exercise program was 79% in the Kinesio taping group and 81% in the foam roller group, with no significant between-group difference (*p* = 0.072). The mean age of the participants in the foam roller group was 44.30 ± 10.99 years and 43.80 ± 10.15 years in the Kinesio taping group. The groups were comparable in terms of demographic characteristics, as presented in Table 1.

Statistically significant within-group improvements were observed in both the Kinesio taping and foam roller groups. In the Kinesio taping group, significant pre- to post-treatment improvements were observed in all outcome measures (*p* < 0.001 for Visual Analogue Scale, Oswestry Disability Index, Pressure Pain Threshold, Schober Test, and Fingertip-to-Floor Distance; *p* = 0.012 for Pittsburgh Sleep Quality Index; *p* = 0.003 for Tampa Scale for Kinesiophobia). In the foam roller group, significant improvements were observed in Oswestry Disability Index, Visual Analogue Scale, Pressure Pain Threshold, Schober Test, and Fingertip-to-Floor Distance (all *p* < 0.001), whereas no statistically significant changes were observed in Pittsburgh Sleep Quality Index (*p* = 0.146) or Tampa Scale for Kinesiophobia (*p* = 0.063).

Between-group comparisons showed significantly lower post-treatment pain intensity in the foam roller group (*p* = 0.015) and significantly lower post-treatment kinesiophobia in the Kinesio taping group (*p* = 0.009). No significant post-treatment differences were observed between the groups for Oswestry Disability Index (*p* = 0.115), Pressure Pain Threshold (*p* = 0.832), Pittsburgh Sleep Quality Index (*p* = 0.611), Schober Test (*p* = 0.230), or Fingertip-to-Floor Distance (*p* = 0.946). A significant baseline difference was observed only for Oswestry Disability Index (*p* = 0.011).

Analysis of change scores showed a greater reduction in pain intensity in the foam roller group (Δ = −5.10 ± 1.32) than in the Kinesio taping group (Δ = −4.24 ± 1.41), with a between-group mean difference in change of −0.86 (95% CI: −1.57 to −0.15). For kinesiophobia, the mean change was −3.57 ± 6.07 in the Kinesio taping group and −1.07 ± 3.02 in the foam roller group, with a between-group mean difference in change of 2.50 (95% CI: 0.00 to 5.00). No meaningful between-group differences in change were observed for the remaining outcomes, as presented in Table 2.

## 4. Discussion

The study findings indicate that both intervention groups showed significant within-group improvements in functionality, pain intensity, pressure pain threshold, and flexibility following treatment. However, between-group comparisons showed significantly lower post-treatment pain intensity in the foam roller group and lower kinesiophobia in the Kinesio taping group, while no significant between-group difference was observed in sleep quality. These findings suggest that the two interventions may have different effects across pain-related and psychosocial outcomes.

In recent years, conservative therapeutic modalities such as Kinesio taping and foam rolling have attracted increasing attention in the management of non-specific low back pain due to their potential to address not only localized musculoskeletal dysfunction but also broader symptoms such as sleep disturbances, kinesiophobia, and reduced quality of life [40]. Kinesio taping is supported by growing empirical evidence for its role in pain reduction, neuromuscular activation, and functional recovery [41]. Its effects may be related to the tape’s elastic properties, which create a micro-lifting effect on the skin and may stimulate cutaneous mechanoreceptors and modulate pain through mechanisms such as gate control [42]. Additionally, Kinesio taping may enhance local circulation and proprioceptive feedback, improving motor control and postural alignment [6,43]. On the other hand, foam rolling may provide self-myofascial release through mechanical pressure, reducing fascial tension and enhancing tissue flexibility [5]. Its analgesic effects may be associated with improved tissue compliance and modulation of nociceptive input via peripheral and central mechanisms, along with temporary increases in stretch tolerance and pain threshold [5].

The analgesic effects of these therapeutic approaches are not limited to reductions in pain intensity but may also be accompanied by improvements in functional status. Kinesio taping may exert its therapeutic benefits by enhancing proprioceptive input and modulating sensorimotor control, with previous studies reporting significant reductions in pain and improvements in function associated with continuous afferent stimulation and postural support [43]. Consistent with this evidence, the present study demonstrated statistically significant improvements in pain and functional outcomes in the Kinesio taping group. Foam rolling, by contrast, may act primarily through mechanical mechanisms such as myofascial release and enhanced tissue elasticity, which are associated with short-term reductions in discomfort and improvements in function [4,5]. In our findings, the foam rolling group also exhibited significant within-group improvements in pain and function, while post-treatment pain intensity differed significantly between groups in favor of foam rolling. These findings suggest that foam rolling can be a practical component of conservative care, although its proposed mechanisms may differ from the sensorimotor effects associated with Kinesio taping. Both groups showed mean reductions in VAS exceeding the reported 1.4-cm minimum clinically important difference; however, the between-group difference in change (0.86 cm) was smaller than this threshold and should therefore be interpreted cautiously.

Reduced pressure pain threshold (PPT) indicates heightened mechanical pain sensitivity and may also be associated with central sensitization in individuals with non-specific low back pain. Kinesio taping may enhance PPT by providing continuous cutaneous stimulation and proprioceptive input, thereby modulating nociceptive processing. Liu et al. [44] reported significant increases in PPT following Kinesio taping in healthy adults. In contrast, foam rolling may improve PPT through mechanical and neurophysiological mechanisms such as myofascial release, mechanoreceptor desensitization, and increased soft tissue compliance. Khan et al. [45] reported improvements in flexibility following a four-week foam rolling program. Fijavž et al. [4] reported sustained improvements in PPT for up to six months in healthy individuals. Although no significant between-group difference was found in our study, both the Kinesio taping and foam rolling groups exhibited significant within-group increases in PPT over time. This suggests that both interventions may effectively reduce mechanical pain sensitivity through distinct, yet similarly beneficial, mechanisms, although these mechanisms were not directly assessed in the present study.

Reduced flexibility of the lumbar and hamstring muscles is common in individuals with chronic non-specific low back pain, contributing to limited mobility and function. Kinesio taping has been reported to improve flexibility and lumbar mobility in individuals with low back pain [40,41]. Similarly, foam roller has been reported to improve flexibility, potentially through changes in myofascial tissue properties and stretch tolerance [4]. In our study, both Kinesio taping and foam roller significantly improved flexibility, with no significant difference between the groups. While Kinesio taping may act through neuromuscular mechanisms, foam rolling may influence flexibility through changes in tissue properties and stretch tolerance [4,46]. Thus, both methods showed improvements in flexibility, with no significant between-group differences, despite their potentially differing mechanisms.

Kinesio taping has demonstrated promising potential in reducing kinesiophobia, with studies [40] reporting significant improvements that may be associated with enhanced sensorimotor feedback and postural support. Although foam rolling may alleviate pain and improve flexibility, its impact on kinesiophobia remains inconclusive, with previous research reporting no significant between-group differences in kinesiophobia following roller-based myofascial release [13]. Differences between previous findings and the present results may be related to variations in participant characteristics, intervention protocols, treatment duration, and outcome assessment methods. In our study, significant reductions in kinesiophobia were observed exclusively in the Kinesio taping group, whereas no meaningful within-group change was noted in the foam roller group; post-treatment kinesiophobia was also significantly lower in the Kinesio taping group. This finding suggests that Kinesio taping may offer benefits beyond its mechanical effects, potentially through neurosensory input and movement reassurance. In contrast, the predominantly mechanical effects of foam rolling may have a more limited influence on fear of movement. However, these potential mechanisms were not directly assessed in the present study.

Sleep disturbances are frequently associated with chronic non-specific low back pain and may contribute to the overall burden of the condition [14]. In the present study, although a significant improvement in PSQI scores was observed within the Kinesio taping group, this change was not accompanied by a significant difference between the two interventions. It is also noteworthy that baseline PSQI scores in both groups were close to the cut-off for poor sleep quality, which may have limited the extent of improvement observed during the intervention period.

This study has several limitations that warrant consideration. The relatively short follow-up period limits conclusions regarding long-term efficacy, and the sample size may constrain the generalizability of the findings. In addition, the baseline ODI scores indicated that the study population predominantly represented individuals with moderate disability; therefore, the findings may not be generalizable to patients with more severe disability. Larger-scale, multicenter trials with extended follow-up are needed to confirm these results. Future research should also explore the distinct neurophysiological mechanisms of Kinesio taping and foam roller, especially their roles in central pain modulation and psychosocial outcomes such as fear-avoidance and sleep disruption.

Building upon these findings, it is noteworthy that while both Kinesio taping and foam roller demonstrated pain-reducing effects, Kinesio taping showed more favorable findings for kinesiophobia, whereas no significant between-group difference was observed in sleep quality. This distinction suggests that Kinesio taping may provide additional benefits for certain psychological aspects of chronic low back pain alongside its physical effects. Conversely, foam roller may primarily exert mechanical effects, which, although beneficial for muscle tension and flexibility, may have a more limited influence on the complex psychological factors associated with chronic pain. Clinically, these results suggest that incorporating Kinesio taping into rehabilitation programs may be particularly relevant when kinesiophobia accompanies physical discomfort.

The findings support Kinesio taping and foam roller as practical conservative approaches for chronic non-specific low back pain, with the two interventions showing different patterns of improvement across physical and psychosocial outcomes. Kinesio taping’s continuous sensory input may influence motor control and fear of movement, aligning with biopsychosocial pain management frameworks. While practical for targeting flexibility and myofascial tension, foam roller may primarily influence physical outcomes. Together, these results highlight the importance of a tailored, multimodal rehabilitation strategy, with Kinesio taping potentially offering added value when kinesiophobia is a relevant clinical concern.

## 5. Conclusions

This study demonstrates that both Kinesio taping and foam rolling are effective conservative interventions for managing chronic non-specific low back pain, yielding significant within-group improvements in functionality, pain intensity, pressure pain threshold, and flexibility. However, the interventions showed different patterns of response, with lower post-treatment pain intensity observed in the foam roller group and lower kinesiophobia in the Kinesio taping group, while no significant between-group difference was observed in sleep quality. These findings suggest that both interventions may provide clinical benefits, with their relative effects varying across physical and psychosocial outcomes. The distinct mechanisms underlying each intervention may account for these differences. Kinesio taping may exert neurosensory and proprioceptive effects that influence sensorimotor control, postural stability, and pain modulation, which may also contribute to changes in fear-avoidance behaviours. In contrast, foam roller may primarily provide mechanical effects through myofascial release and changes in tissue compliance, which may contribute to improvements in pain and flexibility.

Clinically, these results support the use of both Kinesio taping and foam roller as components of conservative rehabilitation for individuals with non-specific low back pain. The findings suggest that treatment selection may be guided by individual clinical priorities, with foam roller showing favorable findings for pain intensity and Kinesio taping for kinesiophobia. A multimodal, patient-specific approach targeting mobility, muscle function, pain, and psychosocial wellbeing may therefore be appropriate. Future large-scale, longitudinal studies are warranted to further elucidate the neurophysiological and psychosocial mechanisms underlying these effects and to optimize intervention protocols for long-term efficacy.

## Figures and Tables

**Table 1:** Study characteristics and patient demographic details.

Demographic Details	Foam Roller Group (Mean ± SD)	Kinesio Taping Group (Mean ± SD)	*p*
Age (yr)	44.30 ± 10.99	43.80 ± 10.15	0.85
Weight (kg)	76.67 ± 11.20	77.73 ± 13.30	0.91
Height (cm)	168.90 ± 6.13	168.77 ± 5.29	0.73
BMI (kg/m^2^)	26.80 ± 3.13	27.18 ± 3.70	0.67

Abbreviations: BMI = body mass index, SD = standard deviation.

**Table 2:** Within- and Between-Group Comparisons Before and After Treatment.

	KT				FR				Between Groups Difference		
	Before Treatment (Mean ± SD)	After Treatment (Mean ± SD)	Δ (Mean ± SD)	*p*	Before Treatment (Mean ± SD	After Treatment (Mean ± SD)	Δ (Mean ± SD)	*p*	Before Treatment *p*	After Treatment *p*	Difference in Δ (95% CI)
ODI (%)	30.30 ± 7.53	19.43 ±4.68	−10.87 ± 6.54	<0.001 *	34.77 ± 5.42	21.17 ± 3.66	−13.60 ± 5.05	<0.001 *	0.011 *	0.115	−2.73 (−5.75 to 0.29)
VAS (cm)	7.57 ± 0.97	3.33 ± 1.18	−4.24 ± 1.41	<0.001 *	7.77 ± 0.86	2.67 ± 0.84	−5.10 ± 1.32	<0.001 *	0.402	0.015 *	−0.86 (−1.57 to −0.15)
PPT (kg/cm^2^)	5.80 ± 1.05	6.86 ± 1.50	+1.06 ± 1.27	<0.001 *	5.83 ± 0.91	6.92 ± 0.84	+1.09 ± 0.47	<0.001 *	0.896	0.832	0.03 (−0.47 to 0.53)
Schober Flexibility Test (cm)	13.76 ± 0.97	16.21 ± 0.93	+2.45 ± 1.10	<0.001 *	13.79 ± 1.74	15.74 ± 1.88	+1.95 ± 0.86	<0.001 *	0.935	0.230	−0.50 (−1.01 to 0.01)
Fingertip-to-Floor Distance Test (cm)	6.68 ± 1.79	3.16 ± 1.30	−3.52 ± 1.62	<0.001 *	6.63 ± 2.16	3.13 ± 1.69	−3.50 ± 1.41	<0.001 *	0.933	0.946	0.02 (−0.77 to 0.81)
PSQI (score)	5.73 ± 3.81	4.63 ± 2.87	−1.10 ± 2.23	0.012 *	5.43 ± 2.91	5 ± 2.68	−0.43 ± 1.58	0.146	0.733	0.611	0.67 (−0.33 to 1.67)
TSK (score)	43.10 ± 7.11	39.53 ± 5.16	−3.57 ± 6.07	0.003 *	43.5 ± 3.05	42.43 ± 2.75	−1.07 ± 3.02	0.063	0.778	0.009 *	2.50 (0.00 to 5.00)

Data are presented as mean ± standard deviation (SD). Δ represents the change from baseline to post-treatment (post-treatment − baseline). Within-group comparisons were performed using paired-samples Student’s *t*-tests, and between-group comparisons at baseline, post-treatment, and for change scores were performed using independent-samples Student’s *t*-tests. Between-group differences in change scores are presented as mean differences with 95% confidence intervals (CIs). * *p* < 0.05 was considered statistically significant. ODI = Oswestry Disability Index (%); VAS = Visual Analogue Scale (cm); PPT = Pressure Pain Threshold (kg/cm^2^); FTF = Fingertip-to-Floor Distance Test (cm); PSQI = Pittsburgh Sleep Quality Index (score); TSK = Tampa Scale of Kinesiophobia (score).

## Data Availability

The data presented in this study are available from the corresponding author upon reasonable request. The data are not publicly available due to privacy and ethical restrictions involving human participants.

## Supplementary Materials

The following supporting information can be downloaded at: https://www.mdpi.com/article/10.3390/healthcare14172853/s1, CONSORT 2025 checklist of information to include when reporting a randomised trial.
